# Galactosylated Polymer/Gold Nanorods Nanocomposites for Sustained and Pulsed Chemo-Photothermal Treatments of Hepatocarcinoma

**DOI:** 10.3390/pharmaceutics14112503

**Published:** 2022-11-18

**Authors:** Gaetano Giammona, Salvatore Emanuele Drago, Giovanna Calabrese, Paola Varvarà, Maria Giovanna Rizzo, Nicolò Mauro, Giuseppe Nicotra, Sabrina Conoci, Giovanna Pitarresi

**Affiliations:** 1 Dipartimento di Scienze e Tecnologie Biologiche Chimiche e Farmaceutiche (STEBICEF), Università degli Studi di Palermo, Via Archirafi 32, 90123 Palermo, Italy; 2 Department of Chemical, Biological, Pharmaceutical and Environmental Sciences, University of Messina, Viale Ferdinando Stagno d’Alcontres 31, 98168 Messina, Italy; 3 Istituto per la Microelettronica e Microsistemi, Consiglio Nazionale delle Ricerche (CNR-IMM) 95121 Catania, Italy; 4 Department of Chemistry ‘‘Giacomo Ciamician’’, University of Bologna, Via Selmi 2, 40126 Bologna, Italy

**Keywords:** polyaspartamide, gold nanorods, sorafenib, lenvatinib, nanoparticles, drug delivery

## Abstract

In this paper, we propose a rational design of a hybrid nanosystem capable of locally delivering a high amount of hydrophobic anticancer drugs (sorafenib or lenvatinib) and heat (hyperthermia) in a remote-controlled manner. We combined in a unique nanosystem the excellent NIR photothermal conversion of gold nanorods (AuNRs) with the ability of a specially designed galactosylated amphiphilic graft copolymer (PHEA-g-BIB-pButMA-g-PEG-GAL) able to recognize hepatic cells overexpressing the asialoglycoprotein receptor (ASGPR) on their membranes, thus giving rise to a smart composite nanosystem for the NIR-triggered chemo-phototherapy of hepatocarcinoma. In order to allow the internalization of AuNRs in the hydrophobic core of polymeric nanoparticles, AuNRs were coated with a thiolated fatty acid (12-mercaptododecanoic acid). The drug-loaded hybrid nanoparticles were prepared by the nanoprecipitation method, obtaining nanoparticles of about 200 nm and drug loadings of 9.0 and 5.4% *w*/*w* for sorafenib and lenvatinib, respectively. These multifunctional nanosystems have shown to convert NIR radiation into heat and release charged drugs in a remote-controlled manner. Then, the biocompatibility and synergistic effects of a chemo-phototherapy combination, as well the receptor-mediated internalization, were evaluated by an in vitro test on HepG2, HuH7, and NHDF. The results indicate that the proposed nanoparticles can be considered to be virtuous candidates for an efficient and selective dual-mode therapy of hepatocarcinoma.

## 1. Introduction

Despite the therapeutic avant-garde, hepatocellular carcinoma (HCC) is still a leading cause of death globally [1]. To date, the HCC treatment protocol contemplates surgery for the initial stages and chemotherapy for the advanced stages [2].

The chemotherapeutic treatment of choice involves the use of sorafenib, a multiple kinase inhibitor, available as oral tablets (Nexavar^®^). Nevertheless, the oral administration of sorafenib is associated with systemic toxicity, a low bioavailability, food–drug interactions, and a poor pharmacokinetic performance, as reported by several studies at both preclinical and clinical levels [3,4,5]. In addition to these issues, some sorafenib-treated patients show a reduction in the therapeutic response due to an intrinsic or acquired drug resistance [6].

With the aim of addressing some of sorafenib deficiencies, the anti-angiogenic multi-kinase inhibitor lenvatinib was recently proposed as a first-line therapy of advanced HCC without an invasion of the main portal vein. Actually, lenvatinib exhibited its efficacy in several studies, including phase III clinical trials, demonstrating significant improvements in the clinical parameters such as progression-free survival (PFS) and time to progression (TTP) in comparison with sorafenib [7].

Despite the advances offered by this new active molecule, pharmaceutical research is still focusing on developing new approaches to overcome the limitations of conventional therapy, which mainly involve a poor selectivity and undesired side effects [8,9]. 

Among these, the approaches proposed by nanomedicine appears to be particularly promising. Indeed, although only few nanosystems have been approved for clinical use [10], there is a growing optimism that applications of nanotechnology to medicine will contribute to significant advances in both the diagnosis and treatment of cancer.

The appropriately designed nanosystems offer a unique opportunity to achieve the controlled delivery of anticancer drugs by optimizing the physicochemical properties of the nanometric vehicle.

The first generation of colloidal anticancer drug (DDS) delivery systems was designed to exploit only the EPR effect, resulting in the biodistribution of nanosystems, pharmacokinetic profiles and an accumulation in the tumor [11] by mainly regulating the nanoscale and the properties of the surface. On the other hand, the second generation of colloidal anticancer DDS was designed to selectively deliver the therapeutic agent at the tumor level, saving healthy tissues [12].

In this way, delivery systems decorated with ligands able to be recognized by specific membrane receptors overexpressed by tumor cells have been developed [13].

Among the various possible targets present in HCC cells, the asialoglycoprotein receptor (ASGPR) is of considerable interest as it is widely expressed by hepatocytes and poorly distributed in extrahepatic tissues [14]. The ASGPR is able to recognize and generally bind monosaccharides, disaccharides, and polysaccharides, but also derivatives such as glycoproteins and polymers with saccharide residues. 

Several studies in the literature report how, in particular, the surface modification of nanosystems with galactose residues has significantly increased the internalization of the nanosystems in the tumor target, thanks to the great affinity exhibited towards the ASGPR [15,16,17].

Despite all these considerations, a resistance to chemotherapy treatment in some types of cancer remains an unsolved problem.

An alternative approach that could overcome these limitations is represented by photothermal therapy (PTT), where photo-active agents (such as gold nanoparticles [18], graphene oxide [19], superparamagnetic iron oxide nanoparticles [20], and carbon nanodots [21]) are used, capable of converting the energy of electromagnetic radiation into heat, which causes various cytotoxic effects that ultimately lead to the death of cancer cells [22,23].

In this context, rod-shaped gold nanoparticles (AuNRs) represent a fascinating option due to the excellent photostability and easy tunability of their optical properties during synthesis. Indeed, a careful choice of the synthetic parameters gives them a highly tailored longitudinal localized surface plasmon resonance (LSPR), which is responsible for an NIR excitation and the photothermal capabilities.

In addition, the AuNRs excitation/dissipation properties are remarkably useful because the NIR region is also known as the biological window (700–1000 nm). Here, the absorption of water and hemoglobin (and hence of body tissues) is minimized, enhancing the penetration depth and thus the hyperthermia efficacy [24].

Following this premise, the present work aims to develop a novel hybrid organic/inorganic nanosystem that combines chemotherapy and photothermal therapy for the treatment of HCC.

To achieve this, a new graft copolymer was synthesized starting from α,β-poly(N-2-hydroxyethyl)-D,L-aspartamide (PHEA), a biocompatible water-soluble amino acid-based polymer, whose derivatives have been widely used for drug and gene delivery and biomedical applications [25,26,27,28,29,30,31,32]. 

To confer amphiphilicity and to allow for a nanoparticle formation, PHEA was first functionalized with α-bromoisobutyryl bromide (BIBB), obtaining a “macroinitiator” for the polymerization via atom transfer radical polymerization (ATRP), and butyl methacrylate (ButMA) hydrophobic monomers were thus polymerized, in order to make the final derivative, PHEA-g-BIB-pButMA, suitable for the preparation of nanoparticles [33]. Subsequently, in order to obtain a nanosystem that can be preferentially internalized by hepatocytes, the copolymer was further functionalized with galactose (GAL) residues, using poly(ethylene) glycol (PEG) as a spacer in order to confer a greater galactose exposure on the surface of nanosystems with stealth properties.

The PHEA-g-BIB-pButMA-g-PEG-GAL brush copolymer was then used to prepare superstructures that contain both hydrophobic AuNRs (AuNRs-C_12_) and sorafenib or lenvatinib inside the nanoparticles core, obtaining NIR-sensitive multifunctional nanocomposites for the on-demand delivery of drugs and the photothermal therapy of HCC.

## 2. Materials and Methods

### 2.1. Materials

The materials 2-bromoisobutyryl bromide (BIBB), butyl methacrylate (ButMA), dimethylamine (DMA), dimethylformamide (DMF), methanol (MeOH), copper bromide (Cu^I^Br), 2,2′-bipyridyl (Bpy) and triethylamine (TEA), Bis (4-nitrophenyl) carbonate (BNPC), diethyl ether, 2,4,6-trinitrobenzene sulfonic acid (TNBS), Dulbecco’s phosphate-buffered saline (DPBS), Tween80, NaOH, HCl, HNO_3_ anthrone, sulfuric acid, poly(ethylene glycol) bis(amine)2000 (H_2_N-PEG-NH_2_), lactose, sodium cyanoborohydride, hexadecyltrimethylammonium bromide (CTAB ≥ 96%), silver nitrate, L-ascorbic acid, sodium borohydride, Gold(III) chloride hydrate, and sorafenib tosylate were purchased from Sigma-Aldrich (Italy).

Lenvatinib mesylate was purchased from D.B.A. Italia. The monomethyl ether hydroquinone present in the commercially available ButMA (used as a stabilizing agent), was removed through a basic activated aluminium oxide column.

α, β-poly (N-2-hydroxyethyl)-D,L-aspartamide (PHEA) was obtained through total aminolysis of polysuccinimide (PSI) with ethanolamine in a DMF solution and purified according to the previously reported procedure [34]. The spectroscopic data corroborate the polymer structure.

^1^H-NMR (300 MHz, D_2_O, 25 °C, TMS): δ 2.71 (m, 2H PHEA, –COCHC**H_2_**CONH–), δ 3.24 (m, 2H PHEA, –NHC**H_2_**CH_2_O–), δ 3.55 (m, 2H PHEA, –NHCH_2_C**H_2_**OH), δ 4.59 [m, 1H PHEA, –NHC**H**(CO)CH_2_–].

### 2.2. Cell Culture

The human HCC cell lines (HuH7 and HepG2) and human dermal fibroblasts (NHDF) were furnished by Merck (Italy). The cells were cultured as adherent monolayers in Dulbecco’s Modified Eagle’s medium (DMEM) (EuroClone), supplemented with 100 U/mL of penicillin, 10% fetal bovine serum (FBS) (Gibco), 100 g/mL of streptomycin, 0.6 g/mL of amphotericin B (Merck, Milan, Italy), and 2 mM of l-glutamine (EuroClone) and they were grown in a humidified atmosphere of 5% CO_2_ in the air at 37 °C. The medium was replaced twice a week and the cells were divided at about 80% of confluence.

### 2.3. General Procedure for the Derivatization of PHEA with 2-Bromoisobutyryl Bromide

The PHEA-g-BIB copolymer was synthesized according to a procedure already reported [26]. Briefly, 500 mg of PHEA (3.1 mmol of OH) was dissolved in 10 mL of aDMA at room temperature, and then 440 µL of TEA were added (molar ratio between TEA and PHEA hydroxyl groups equal to 1). Then, the reaction mixture was cooled to 0 °C and 390 µL of BIBB were added (molar ratio between BIBB and PHEA hydroxyl groups equal to 1). The reaction mixture was left at room temperature for 4 h and then dropped into cold diethyl ether. The precipitate was washed twice in the same solvent, desiccated at a low pressure, dialyzed against distilled water (MWCO 12–14 kDa), and freeze dried.

^1^H-NMR (300 MHz, DMF, 25 °C) δ: 1.95 (s, 6H BIB, (C**H_3_**)_2_–C–CO–), 3.29 (m, 2H PHEA, –NHC**H_2_**CH_2_O–), 3.58 (m, 2H PHEA, –NHCH_2_C**H_2_**OH), 4.25 (m, 2H PHEA, –NHCH_2_C**H_2_**O–BIB), 4.76 (m, 1H PHEA –NHC**H**(CO)CH2–).

### 2.4. General Procedure for the Derivatization of PHEA-BIB with Poly (Butyl Methacrylate) through ATRP

The PHEA-g-BIB-pButMA copolymer was obtained as reported elsewhere [33]. A total of 150 mg of PHEA-g-BIB (corresponding to 0.255 mmol of Br) were dissolved in a previously degassed 1:1 DMF/MeOH (*v*/*v*) mixture (12 mL), followed by the addition of 400 µL of butylmethacrylate (molar ratio between ButMA monomer and Br equal to 10). Later, under vigorous stirring and argon bubbling, 35.8 mg of Cu^I^Br catalyst (molar ratio between Cu^I^Br and Br in polymer side chain equal to 1) and 156.18 mg of bipyridine (Bpy) (molar ratio between Bpy and Cu^I^Br equal to 4) were mixed with a spatula and then added. The reaction mixture was maintained under an argon atmosphere at 50 °C for 20 h, and then, by opening the reaction flask, stopped.

The polymer was then Isolated by dripping the reaction mixture into a water/MeOH mixture (1:1) and the precipitate was washed twice in the same mixture. The white residue was dissolved in DMF, purified by exhaustive dialysis (MWCO 12–14 kDa), and freeze dried.

^1^H-NMR (300 MHz, DMF, 25 °C, TMS) δ: 0.99 (m, 6H ButMA, –CH–C**H_3_** and –CH_2_–CH_2_–C**H_3_**), 1.48 (m, 2H ButMA, –CH_2_–C**H_2_**–CH_3_) 1.66 (m, 2H ButMA, –C**H_2_**–CH_2_–CH_3_), 1.90 (s, 6H BIB, (C**H_3_**)_2_–C–CO–), 3.30 (m, 2H PHEA, –NHC**H_2_**CH_2_O–), 3.58 (m, 2H PHEA, –NHCH_2_C**H_2_**OH), 4.13 (m, 2H ButMA, (CO)–O–C**H_2_**–CH_2_–CH_2_), 4.74 (m, 1H PHEA –NHC**H**(CO)CH_2_–).

### 2.5. Introduction of Lactose end Group onto Poly(Ethylene Glycol) bis(Amine) (H_2_N-PEG-NH_2_)

The O-(2-aminoethyl)-O′-galactosyl polyethylene glycol (H_2_N-PEG-GAL) derivative was synthesized as reported elsewhere [17,35]. Briefly, 800 mg of H_2_N-PEG-NH_2_ (0.8 mmol of NH_2_) was dissolved in 16 mL of borate buffer with a pH value of 9 and 62.8 mg of sodium cyanoborohydride (0.5 M in borate buffer at a pH of 9), and 342.4 mg of lactose (0.5 M in a borate buffer at a pH of 9) were added sequentially.

Then, the reaction mixture was left at 40 °C for 24 h, then dialyzed against distilled water (Spectra/Por^®^ Standard RC tubing; MWCO 1 kDa) for 24 h and lyophilized.

The content of the amine-terminated chains was quantified by a TNBS assay [36], while the content of galactose-terminated chains was quantified by the anthrone assays [17].

### 2.6. Synthesis of PHEA-g-BIB-pButMA-g-PEG-GAL Copolymer

The derivatization of PHEA-g-BIB-pButMA with H_2_N-PEG-GAL was performed by using the coupling agent Bis(4-nitrophenyl) carbonate (BNPC).

During the first step, 200 mg of PHEA-g-BIB-pButMA (0.29 mmol repeating unit) were dissolved in 4 mL of aDMF, then 400 µL of BNPC (20.4 mg mL^−1^ in aDMF) were added (molar ratio between BNPC and repeating unit equal to 0.08). The reaction mixture was left under stirring at 40 °C for 4 h.

Later, 91.45 mg of H_2_N-PEG-GAL (corresponding to 50.35 mg of pure H_2_N-PEG-GAL) dissolved in 2 mL of a-DMF were added to the reaction mixture.

The reaction mixture was maintained under stirring at 25 °C overnight and then dialyzed (MWCO 12–14 kDa) for two days against basic water (NaOH) and for three more days against ultrapure water; after that, the solution was freeze dried and stored for a further characterization. 

^1^H-NMR (300 MHz, DMF, 25 °C, TMS) δ: 0.99 (m, 6H ButMA, –CH–C**H_3_** and –CH_2_–CH_2_–C**H_3_**), 1.48 (m, 2H ButMA, –CH_2_–C**H_2_**–CH_3_) 1.66 (m, 2H ButMA, –C**H_2_**–CH_2_–CH_3_), 1.90 (s, 6H BIB, (C**H_3_**)_2_–C–CO–), 3.29 (m, 2H PHEA, –NHC**H_2_**CH_2_O–), 3.58 (m, 172H PEG, –OC**H_2_**C**H_2_**O–), 4.13 (m, 2H ButMA, (CO)–O–C**H_2_**–CH_2_–CH_2_), 4.74 (m, 1H PHEA –NHC**H**(CO)CH_2_–).

### 2.7. Size Exclusion Chromatography

The weight-average molecular weight (Mw) and polydispersity (PD) of each copolymer were determined by a size exclusion chromatography (SEC) analysis, performed using Phenomenex Phenogel 5u 10^4^A columns connected to an Agilent 1260 Infinity Multi-Detector GPC/SEC system (Milan, Italy). Analyses were carried out at 50 °C in DMF + 0.1 M LiBr as the mobile phase with a flow of 0.8 mL/min, performing an absolute calibration of the instrument with a polystyrene standard (70 kDa).

### 2.8. Synthesis of CTAB-AuNRs

The preparation of gold nanorods (AuNRs) was carried out following a silver-assisted seed-mediated growth procedure in CTAB [18]. The gold seeds were prepared by the rapid reduction of the Au^III^ ions carried out by NaBH_4,_ by mixing 25 μL of 0.05 M HAuCl_4_ solution with CTAB 0.1 M (4.7 mL), followed by the addition of 300 μL of NaBH_4_ 0.01 M, under vigorous stirring. Simultaneously, to 50 mL of CTAB 0.1 M, 500 μL of 0.05 M HAuCl_4_ solution and 950 μL of HCl 1 M were added and left to equilibrate for a few minutes. Then, of 600 μL of AgNO_3_ 0.01 M, 400 μL of ascorbic acid 0.1 M and 120 μL of freshly prepared seeds were added in sequence, leading to the CTAB-AuNRs formation.

### 2.9. Transmission Electron Microscopy (TEM) Analyses

The CTAB-AuNRs dispersion was washed 2 times with 2 mL of ultrapure water and recovered by centrifugation. Subsequently, the pellet was dispersed in 2 mL of ultrapure water and a few microliters of AuNRs were deposited onto lacey carbon grids and left to dry in the air before the examination. The observation was performed by transmission electron microscopy (TEM) analysis using the bright field in a conventional parallel beam (CTEM) mode (BF). An ATEMJEOL JEM-2010 (JEOL Ltd., Musashino, Akishima, Tokyo, Japan) equipped with a 30 mm^2^ window energy-dispersive X-rays spectrometer was used.

### 2.10. Preparation of Coated AuNRs (AuNRs-C_12_)

The coating of AuNRs with 12-mercaptododecanoic acid (12-MDA) was carried out by exploiting the well-known thiol-gold chemistry [37]. Briefly, 200 mL of CTAB-AuNRs were washed 2 times with 200 mL of ultrapure water and recovered by centrifugation (15,000 rpm, 60 min, and 25 °C). Subsequently, the pellet was dispersed into 6 mL of water and 6 mL of 12-MDA in DMSO were added to the dispersion of washed AuNRs, following the Au:12-MDA weight ratio equal to 1:5. The dispersion was thus sonicated for 30 min and kept incubating in an orbital shaker for 30 min at 37 °C. Subsequently, 170 mL of DMSO were added and AuNRs-C_12_ recovered by centrifugation (15,000 rpm, 90 min, and 25 °C). The AuNRs-C_12_ were retrieved with 10 mL of DMSO, sonicated for 15 min, and stored at room temperature.

The Au concentration of CTAB-AuNRs and AuNRs-C_12_ was calculated by UV–VIS analysis by measuring the absorbance of the sample at the fixed wavelength of 400 nm [33].

### 2.11. Characterization AuNRs-C_12_

The optical properties of AuNRs and AuNRs-C_12_ in DMSO were evaluated by a double beam spectrophotometer (Shimadzu UV-2401PC) in the range of 350–900 nm. AuNRs-C_12_ and 12-MDA were analyzed by FT-IR spectroscopy (Bruker Alpha II spectrometer) in KBr pellets. In particular, to obtain a solid sample of AuNRs-C_12_, the organic dispersion was dialyzed (MWCO 12–14 kDa) for two days against ultrapure water and then freeze-dried. The FT-IR spectra were recorded in the range of 400–4000 cm^−1^ (scan times: 24; resolution: 4 cm^−1^).

The elemental analysis was carried out with SEM Phenom XL ProX, a scanning electron microprobe.

### 2.12. Preparation of Sorafenib-Loaded Nanoparticles (SOR-NPs) and Lenvatinib-Loaded Nanoparticles (LEN-NPs)

Sorafenib-loaded nanoparticles were prepared by the direct nanoprecipitation method [25].

A suitable amount of AuNRs-C_12_ dispersion corresponding to 2 mg of Au was recovered by centrifugation (10,000 rpm, 10 min, 25 °C); after centrifugation, the supernatant was discarded and the pellet was retrieved with 1 mL of DMF. Subsequently, 500 µL of PHEA-g-BIB-pButMA-g-PEG-GAL solution in DMF (20 mg mL^−1^) and 500 µL of sorafenib tosylate solution in DMF (4.57 mg mL^−1^) were added in sequence. 

The organic dispersion was sonicated for 15 min and then was dripped to 20 mL of PVP aqueous solution (0.5 mg mL^−1^) under continuous stirring (500 rpm). The mixture was left under stirring for 1 h.

The dispersion then was dialyzed (MWCO 12–14 kDa) at room temperature against 2000 mL of ultrapure water for 24 h, replacing the external aqueous phase each 3 h. 

Then, the colloidal dispersion was finally filtered through a 5 μm membrane filter (Sartorius, Minisart Syringe Filter, Göttingen, Germany) and freeze-dried.

Lenvatinib-loaded nanoparticles were prepared by the modified nanoprecipitation method. The organic solution was prepared as described above, replacing 500 µL of sorafenib tosylate solution with 500 µL of lenvatinib mesylate solution at the same concentration.

The organic solution was swirled and 20 mL of ultrapure water were added with a flow rate of 10 mL min^−1^.

The mixture was thus again stirred for 1 h and then the dispersion was ultracentrifuged (40,000 rpm, 60 min, and 15 °C), the pellet was washed with 20 mL of ultrapure water and ultracentrifuged in the same manner. The solid residue was retrieved with 10 mL of PVP aqueous solution (1 mg mL^−1^) and freeze dried.

### 2.13. Dynamic Light Scattering (DLS) and Zeta-Potential Measurements

The size distribution was evaluated by DLS measurements using a Malvern Zetasizer NanoZS instrument equipped with a 632 nm laser with a fixed scattering angle of 173°. The analyses were performed on 1 mL of nanoparticles dispersion in water (0.25 mg mL^−1^). The Z-average and PDI values were obtained from the analysis of the correlograms.

Zeta-potential analysis was performed by aqueous electrophoresis measurements at 25 °C, using the same apparatus for the DLS measurements, on 1 mL of nanoparticles dispersion in water (0.25 mg mL^−1^). The zeta-potential values (mV) were calculated from electrophoretic mobility using the Smoluchowski relationship.

### 2.14. Scanning Electron Microscopy (SEM) Analyses

For morphological studies, a few drops of each liquid dispersion were deposited on a silicon wafer and after the evaporation of the water, the residuals were observed by using a Crossbeam 340 field emission scanning electron microscope (Carl Zeiss Microscopy, Oberkochen, Germany). The SEM analysis was done with an acceleration voltage (EHT) of 3 kV and by detecting type II secondary electrons (SE2). 

### 2.15. Determination of Drug and Gold Content into NPs

The drug content into NPs was quantified using Agilent 1260 Infinity high-performance liquid chromatography (HPLC), equipped with a multiple wavelength detector, operating at 266 nm (for sorafenib) and 240 nm (for lenvatinib), and an Open Lab Chemstation software. The elution was carried out isocratically at 25 °C, using a reverse-phase Gemini C6-phenyl 110A column (Phenomenex 5 μm, 250 mm× 4.60 mm) and methanol/water 85:15 mixture as the eluent with a flow rate of 1 mL min^−1^.

The NPs were dissolved in a known amount of DMF and then methanol was added; the mixture was vigorously stirred for 3 h to extract the drug. The resulting suspension was centrifuged (14,500 rpm for 10 min at 25 °C) and 50 μL of the supernatant were injected.

The resulting chromatograms were analyzed comparing the peaks corresponding to the drug amount loaded into the NPs with a calibration curve obtained with drug solutions standards in the methanol (0.5–100 μg mL^−1^). The drug loading (DL%) was expressed as the weight percent ratio between the amount of loaded drug and the amount of weighted freeze-dried NPs.

The entrapment efficiency (EE%) was expressed as the weight percent ratio between the amount of loaded drug and the initial amount drug used for the nanoparticles’ preparation.

The amount of gold into NPs was determined after a complete oxidation of the samples’ aqueous dispersions in an HCl 37%/HNO_3_ 69.5% 3:1 *v*/*v* mixture, using Spectroquant^®^ Gold Test (Merck, Milan, Italy) and expressed as the weight percent ratio between the amount of loaded gold and the amount of weighted freeze-dried NPs.

### 2.16. Thermal Analysis

Both SOR-NPs and LEN-NPs were also analyzed by differential scanning calorimetry (DSC) coupled with a thermal gravimetric analysis (TGA), using a DSC/TGA 131 EVO (by SETARAM Instruments). Each measurement was performed under the nitrogen atmosphere with a flow of 1 mL min^−1^, using about 5 mg of dried sample placed into an alumina crucible. The samples were heated starting from 20 °C up to 500 °C, with a heating rate of 10 °C min^−1^.

### 2.17. Drug Release Studies

For the drug release experiments, 2.8 mg of SOR-NPs (corresponding to 0.252 mg of sorafenib tosylate) or 2.4 mg of LEN-NPs (corresponding to 0.125 mg of lenvatinib mesylate) were dispersed in 1 mL of DPBS with a pH of 7.4 and placed into a dialysis tubing (12–14 kDa cut-off).

The external media (9 mL) were DPBS for LEN-NPs and DPBS containing 1% (*v*/*v*) of Tween 80.

At fixed time intervals, 0.2 mL of the receiving compartment was withdrawn and replaced with an equal volume of the fresh medium. 

The drug released was quantified using HPLC analysis, as described above.

The photothermal-triggered drug release profiles were evaluated by irradiating the dispersion of nanoparticles, using a GBox diode NIR laser (λ: 810 nm) with a power of 0.7 W mL^−1^ for 5 min or 20 min at different scheduled time intervals (0 h, 1 h, 3 h, and 6 h).

The same experiment set-up was then reproduced to record the thermal rise and subsequent temperature decrease in the external medium under the chosen experimental conditions. In this case, the temperatures were recorded at set time intervals during the laser treatment (for 5 or 20 min) and at the end of the irradiation, until the initial temperature of 37 °C was reached. The temperature was recorded using a fiber optic termometer (±1 °C sensitivity) at a preset laser exposure time and hyperthermia/dissipation profiles were thus obtained by plotting the temperatures versus the exposure time.

### 2.18. Erythrocompatibility Studies

Healthy erythrocytes were collected by centrifuging 8 mL of fresh venous blood at 500× *g* for 10 min and by washing 5 times the pellet retrieved with PBS (pH 7.4) until a colorless supernatant was observed. The pellet was recovered with PBS up to 8 mL and 1 mL of this suspension was further diluted 50 times.

Subsequently, 1.425 mL of the erythrocytes suspension was mixed with 75 µL of SOR-NPs dispersion (1.06 mg mL^−1^) or LEN-NPs dispersion (3.02 mg mL^−1^). Equivalent amounts of free drugs sorafenib tosylate and lenvatinib mesylate were also tested and used as the control experiments.

The mixtures were incubated at 37 °C for 1 h, centrifuged at 500× *g* for 10 min, and the amount of hemoglobin released following erythrolysis was quantified by a UV spectrophotometer at 570 nm. All the data were compared using Triton X-100 as a positive control.

### 2.19. Anticancer Activity of the SOR-NPs and LEN-NPs with and without NIR Exposure

The anticancer activity of the SOR-NPs and LEN-NPs was assessed by an MTS assay using two HCC cell lines (HepG2 and HuH7) and human dermal fibroblasts (NHDF).

In particular, the cells were seeded in a 96-well plate with a density of 1.0 × 10^4^ cells per well (200 μL) and grown for 24 h in a DMEM.

Successively, the medium was replaced with a dispersion of SOR-NPs (sorafenib tosylate concentration ranging from 7.5 to 0.5 μM) or LEN-NPs (lenvatinib mesylate concentration ranging from 15 to 1 μM) and incubated for 24 or 48 h.

After this time, the media containing NPs were removed from each well, the cells were washed twice with DPBS pH 7.4, and 120 μL of MTS assay solution diluted 6-fold in the DMEM was added in each well.

The cells were thus incubated for an additional time (4 h for HepG2 and 2 h for both HUH7 and NHDF) at 37 °C and 5% CO_2_ before reading the absorbance at 492 nm using a Microplate Reader (Multiskan Ex, Thermo Labsystems, Finland). The cell viability was expressed as a percentage of the living cells with respect to the untreated control of the seeded cells (taken as 100%).

In parallel, the combination effect of NIR triggered hyperthermia and drug local release was investigated by exposing all the cell lines incubated with increasing amounts of nanoparticles to an 810 nm diode laser for 300 s (for SOR-NPs P = 10 W, for LEN-NPs P = 6 W). 

At the end, the wells were washed with DPBS pH 7.4 twice and the MTS assay was performed as described above. 

The anticancer effect of the nanoparticles was compared with that of free drugs and empty nanoparticles at equivalent concentrations, treated and untreated with an NIR laser. All the experiments were performed in triplicate.

### 2.20. Cell Uptake Studies

To evaluate the internalization efficiency after the incubation with the cells, the PHEA-BIB-pButMA was at first labelled with rhodamine B. In brief, RhB was dissolved in aDMF (0.128 mg RhB in 0.2 mL) and activated by adding a solution of CDI in aDMF (mol CDI/mol RhB =1.2) maintaining the reaction flask 4 h at 40 °C. Separately, 20 mg of PHEA-g-BIB-pButMA (0.0268 mmol of UR) were dissolved in aDMF (0.5 mL) and, after the addition of TEA (mol TEA/mol RhB = 1.2), the previously activated RhB was dripped into the reaction flask and left to react for 48 h at 40 °C. The product was then precipitated in diethyl ether, washed with ethanol, and purified through dialysis (MWCO 12–14 KDa) after a dissolution in DMF. Finally, the labelled copolymer was functionalized with H_2_N-PEG-GAL, purified, and retrieved following the same steps described for the unlabeled derivative, and used to produce labelled empty NPs by a nanoprecipitation technique in the presence of AuNRs-C_12_ dispersion (without drug) as described above.

The uptake studies were thus carried out on HepG2 and HuH7 and NHDF by seeding each cell line into 2 chambers coverglass systems with a density of 5 × 10^4^ cell per chamber, allowing the cell to adhere and grow for 24 h in supplemented DMEM. The medium was then replaced with fresh dispersions of labelled empty NPs (0.05 mg mL^−1^) and incubated for 4 h or 24 h. At the end, the camber content was discarded, the cells were fixed with formaldehyde solution (4% in DPBS, 5 min, RT), permeabilized with Triton X-100 (0.1% in DPBS,10 min, RT), and stained with phalloidin-FITC (1 h, 37 °C) and 4′,6-diamidino-2-phenylindole (DAPI) (10 min, RT) to visualize the cytoskeleton and nuclei, respectively, washing 3 times with DPBS pH 7.4 before and after each step. Furthermore, a second parallel experiment was performed on the same cell lines pre-incubating the cells with a 250 mM galactose solution for 30 min before incubating the labelled empty NPs for 4 h or 24 h and staining the cells as described above.

The uptake micrographs were acquired by using an Axio Cam MRm (Zeiss) fluorescence microscope equipped with a 100× magnification immersion objective.

### 2.21. Statistical Analysis

All the experiments were repeated at least three times. All the data are expressed as the means ± standard deviation. All the data were analyzed by Student’s *t*-test using Microsoft Excel software.

## 3. Results

### 3.1. Polymer Synthesis and Characterization

The graft copolymer PHEA-gBIB-(pButMA)-g-PEG-GAL was synthesized performing three steps. 

**Scheme 1:**
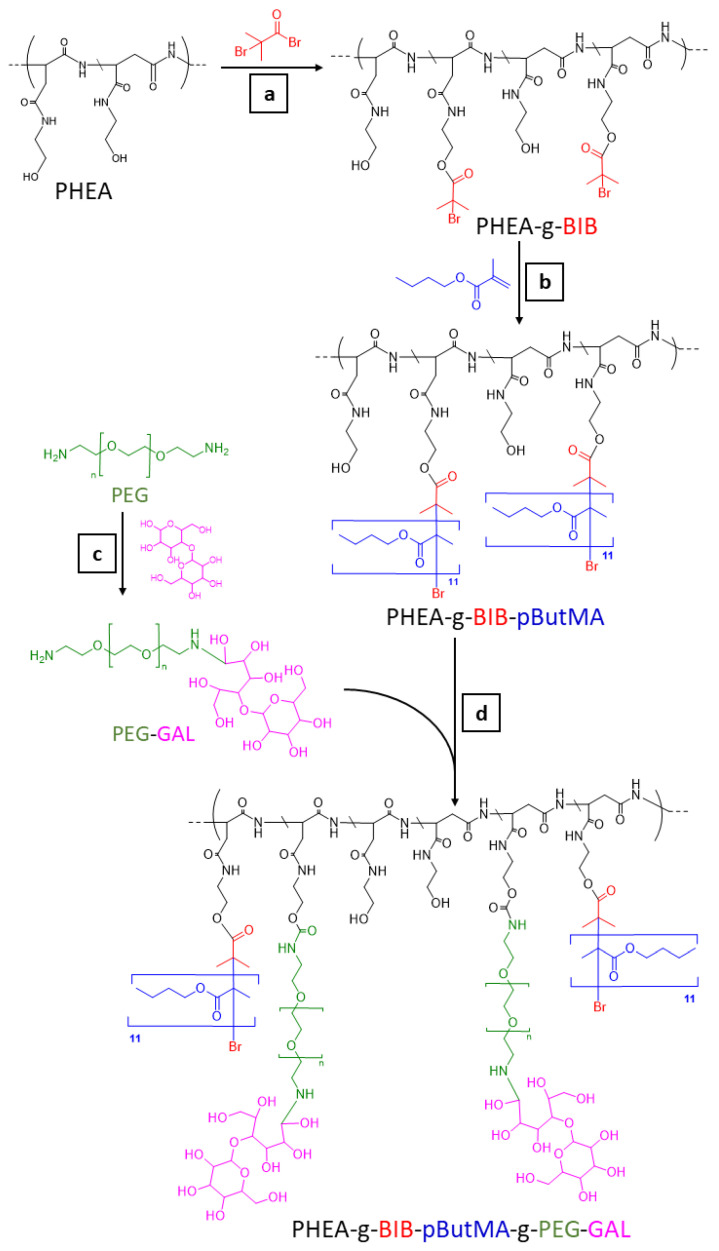
Synthetic pathway of PHEA-g-BIB-pButMA-g-PEG-GAL. (**a**) 1. a-DMA, TEA, 0 °C, 15 min; 2. RT, 4 h. (**b**) Ar atm, DMF/MeOH, Cu^I^Br, Bpy, 50 °C, 20 h. (**c**) Borate buffer 0.5 M pH 9, NaBH_3_CN, 40 °C, 24 h. (**d**) 1. a-DMF, BNPC, 40 °C, 4 h; 2. 25 °C, overnight.

In the first step, the PHEA was derivatized with 2-bromoisobutyryl bromide (BIBB) residues, exploiting the reactivity of the hydroxyl groups towards acyl bromide, using triethylamine as a catalyst (Scheme 1, step a). The degree of derivatization in BIB obtained was equal to 35 mol%. This value was calculated by the ^1^H-NMR (Figure S1) spectrum comparing the integral of the peak attributable to methyl groups at δ 1.95 of BIB with that assigned of CH_2_ of the PHEA backbone at δ 3.29 ppm. 

The obtained multifunctional macroinitiator was thus used as a starting product to carry out the atom transfer radical polymerization (ATRP) of the hydrophobic monomer butyl methacrylate (ButMA), which represent the second reaction step (Scheme 1, step b).

By analyzing the ^1^H-NMR (Figure S2) spectrum of the brush copolymer retrieved, a derivatization degree in ButMA of 385 mol% was calculated, comparing the integral peak attributable to the methyl groups at δ 0.99 of ButMA with that assigned to CH_2_ of the PHEA backbone at δ 3.30 ppm. Considering that the calculated degree of derivatization in BIB is equal to 35 mol%, the extension of the polymerization (n) process of ButMA resulted to be 11 (n = derivatization degree in ButMA/derivatization degree in BIB).

Aiming pendant galactose moieties were then introduced. To do this, O-(2-aminoethyl)-O′-galactosyl polyethylene glycol was synthesized by a reductive amination between bis(amine) polyethylene glycol and lactose (Scheme 1, step c). In more detail, considering that, for the sugars, the equilibrium ratio of aldehyde to lactol widely favors the latter, a galactosylated polyethylene glycol synthesis was performed using a large excess of lactose in order to increase the conversion yield [38].

By a TNBS assay, the amine terminated content was found to be equal to 0.234 mmol/g, while the content of galactose, determined by the anthrone assays, was found to be equal to 0.569 mmol/g. This information suggests that the final product corresponds to a mixture of mono-galactosylated PEG and di-galactosylated PEG, equal to 55% and 45% by weight, respectively.

Although the high amount of sugar could be responsible for the double functionalization of PEG, the formation of this derivative does not interfere with the subsequent reaction step since its reactivity is negligible.

Finally, in the last synthetic phase, the graft copolymer PHEA-g-BIB-pButMA was further derivatized with the proper amount of O-(2-aminoethyl)-O′-galactosyl polyethylene glycol, exploiting the reactivity of the free amino group towards the carbonate ester (Scheme 1, step d). By the ^1^H-NMR spectrum (Figure S3), comparing the integral peak attributable to the methylene groups at δ 3.58 of the PEG with that assigned to CH_2_ of the PHEA backbone at δ 3.29 ppm. The derivatization degree in the PEG resulted to be 7 mol%.

All the obtained copolymers were characterized by an SEC analysis in terms of the weight-average molar weight (Mw) and polydispersity (Mw/Mn)) and the obtained values are reported in Table 1.

### 3.2. Preparation and Characterization of Coated AuNRs

CTAB-assisted gold nanorods synthesis leads to the formation of very stable gold cylindrical nanoparticles. 

**Figure 1:**
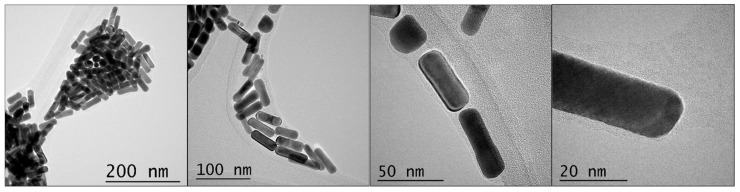
TEM acquisitions on CTAB-AuNRs using different magnifications.

The TEM inspection on CTAB-AuNRs show a rod-shaped nanostructure with a nanometric size featured by an average length of 50 nm and an average diameter of 15 nm (Figure 1).

The AuNRs obtained through this synthetic procedure are hydrophilic and incompatible with the hydrophobic core of the polymeric nanoparticles. To allow for the encapsulation of AuNRs within the core of the nanoparticles, the AuNRs surface was modified with 12-mercaptododecanoic acid (12-MDA), thus obtaining coated hydrophobic AuNRs. 

**Figure 2:**
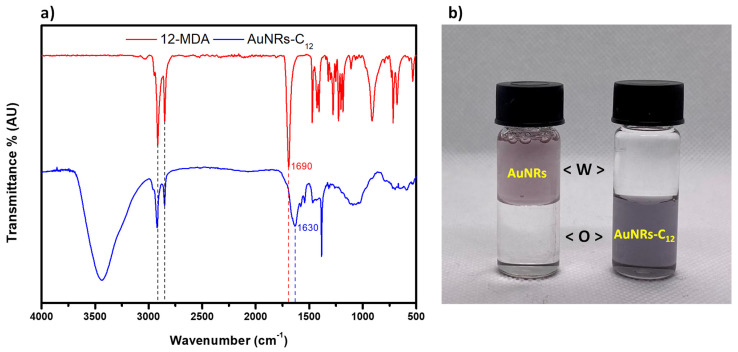
AuNRs hydrophobic coating analyses. (**a**) FT-IR spectra of 12-MDA (red) and 12-MDA-coated AuNRs (blue). (**b**) Visual inspection of the O/W (CHCl_3_/H_2_O) partition equilibria of native AuNRs (**left**) and AuNRs-C_12_ (**right**).

FT-IR analysis corroborated the presence of 12-MDA, confirming the success of the coating procedure. Indeed, the spectrum of AuNRs-C_12_ (Figure 2a) showed both typical bands of C-H and COOH stretching, highlighting however a slight shift of the latter due to a change in the dipole moment after the conjugation of the 12-MDA on a metal surface with a high electron density [39].

The effective hydrophobization was confirmed by a visual inspection of the different partitions equilibria of AuNRs and AuNRs-C_12_ in an O/W biphasic system (CHCl_3_/H_2_O). As shown in Figure 2b, coating AuNR with 12-MDA allows for an increase in the affinity with the CHCl3 phase, reducing the affinity with the aqueous phase. This result has particularly relevant implications because a greater affinity for the hydrophobic phase theoretically guarantees a greater incorporation of AuNRs-C_12_ into the hydrophobic core of the polymer nanoparticles.

**Figure 3:**
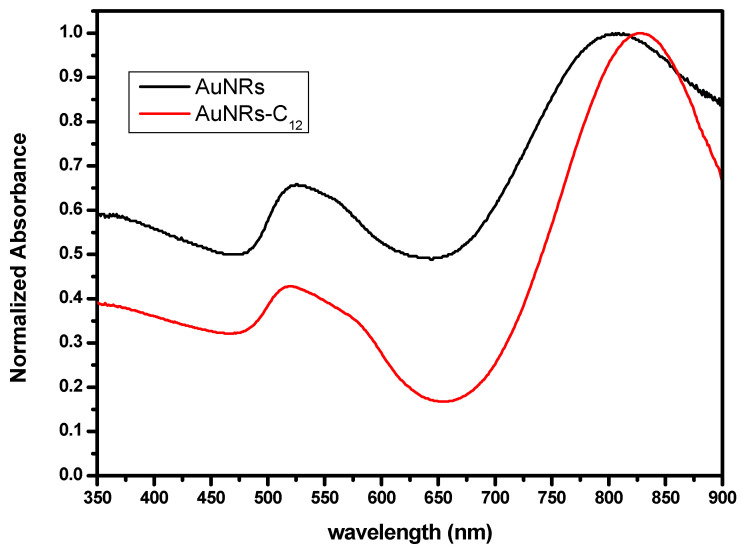
UV spectra of AuNRs (black) and AuNRs-C_12_ (red) dispersed in DMSO.

Coating with 12-MDA consequently allowed a better dispersibility in organic solvents. As further evidence of the improved stability in more hydrophobic environments, the UV-VIS-NIR spectra acquired in an organic solvent (DMSO) of both AuNRs and AuNRs-C_12_ (Figure 3) showed that AuNRs-C_12_ maintain a narrow longitudinal plasmonic resonance peak in the NIR region, while a broadening of the corresponding peak was observed for non-hydrophobized AuNRs, indicating particle aggregation phenomena of the latter.

**Figure 4:**
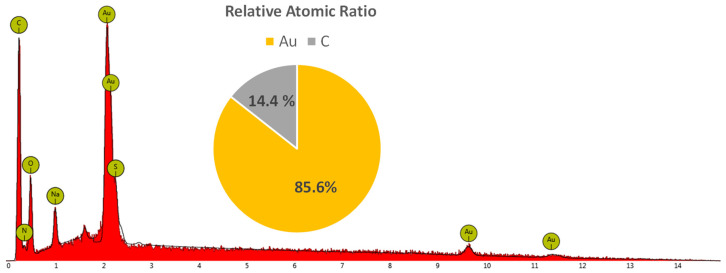
Elemental analysis and relative atomic abundance ratio of gold and carbon on AuNRs-C_12_.

Through elemental analysis, it was possible to determine the quantity of 12-MDA bound to AuNRs. In particular, it was observed that the abundance of gold atoms was about six-fold higher (5.94) than the carbon atoms abundance (Figure 4). 

Furthermore, taking into account that the moles of 12-MDA were equal to one twelfth of those of carbon, it results that the molar ratio between Au and 12-MDA was equal to 71.33.

Therefore, knowing that the dimensions of the AuNRs were 50 × 15 nm, it follows that the coating density was equal to 2.69 chains of 12-MDA per nm^2^.

### 3.3. Preparation and Characterization of AuNRs-C_12_ and Drug-Loaded Polymeric Nanoparticles (SOR-NPs and LEN-NPs)

Since sorafenib and lenvatinib have different chemical–physical properties, different preparation techniques were investigated in order to select the most suitable method, leading to nanoparticles with the best balance between a favorable size, stability in an aqueous medium, and a high drug content.

**Figure 5:**
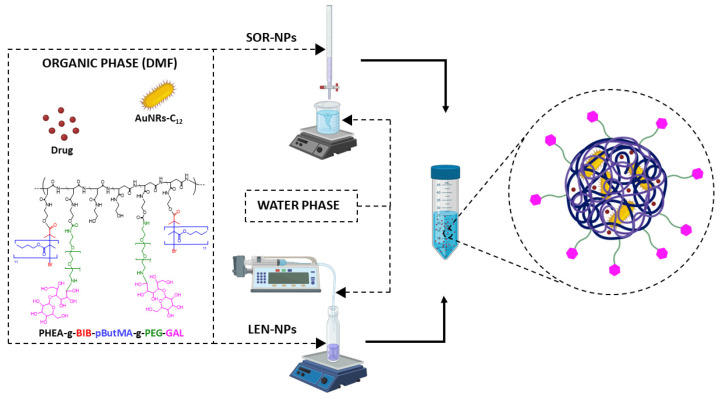
Schematic representation of the preparation of hybrid nanoparticles.

In both cases, the best results were obtained using the nanoprecipitation technique, but the inherent differences in the drugs led to some tailored modifications of the procedure (Figure 5).

In particular, sorafenib-loaded nanoparticles were prepared by dripping the organic solution (containing polymer, sorafenib and AuNRs-C_12_) into an aqueous PVP solution. This latter was chosen because it acts, at the same time, as a stabilizer as well as widely known cryoprotectant, thus not requiring its removal after the nanoparticles’ preparation [40].

In contrast, lenvatinib-loaded nanoparticles were prepared by an “inverted procedure” which consisted of the addition of excess water in the organic solution-containing polymer, lenvatinib and AuNRs-C_12_, under vigorous stirring.

The drug-loaded nanoparticles obtained were analyzed using dynamic light scattering (DLS) to evaluate the size distribution and zeta potential. The weight percentages of sorafenib and lenvatinib entrapped in the particles were calculated by HPLC analysis, while the gold content was evaluated by a colorimetric assay. All the characterizations are summarized in Table 2.

**Figure 6:**
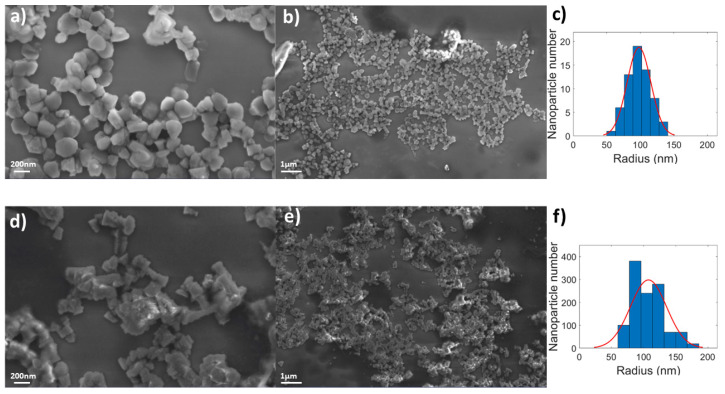
Scanning Electron Microscopy acquisitions (**a**,**b**,**d**,**e**) and dimensional distribution (**c**,**f**) of SOR-NPs (**a**–**c**) and LEN-NPs (**d**–**f**). Scale bars: (**a**) 200 nm; (**d**,**e**) 1 μm.

The SOR-NPs SEM images (Figure 6a,b) revealed the presence of almost spherical nanoparticles with an average radius around 98 ± 18 nm (diameter around 200 nm) with a classical Gaussian distribution (Figure 6c), confirming the dimensions obtained by DLS analysis. In addition, LEN-NPs SEM acquisitions (Figure 6d,e) showed the presence of nanoparticles of a not very regular shape with an average radius around 107 ± 28 nm (diameter around 200 nm) and with a wider Gaussian distribution (Figure 6f) if compared to SOR-NPs, as already indicated by the PDI value of the DLS analysis.

Finally, the DSC analyses (Figures S4 and S5) suggested that the drugs are presumably entrapped into SOR-NPs and LEN-NPs at a molecular level since it was not possible to clearly distinguish the peaks related to the fusion of the free drugs after the incorporation.

### 3.4. Drug Release Studies

The ability of nanoparticles to release the entrapped drug in a controlled manner was evaluated in physiological conditions (pH 7.4 at 37 °C), with and without an NIR laser exposure at a suitable power density. 

**Figure 7:**
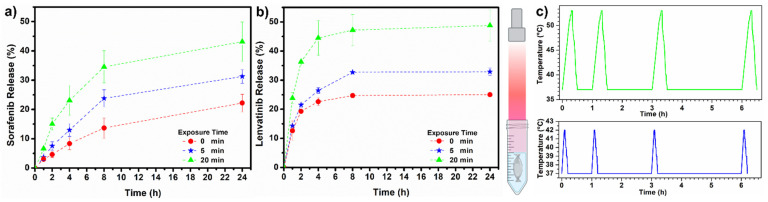
Drug release profiles of (**a**) SOR-NPs and (**b**) LEN-NPs without laser treatment (red) or after 5 min (blue) or 20 min (green) cycles of NIR-laser exposure. (**c**) Thermal rising and temperature decreasing during the 4 irradiation cycles performed with exposure times of 5 min (blue) and 20 min (green).

As shown in Figure 7, both the SOR-NPs and LEN-NPs allow a slow and sustained drug release profile over time under physiological conditions, releasing less than 25% of the entrapped drug in 24 h without recording any potentially problematic burst effect. On the other hand, the NIR irradiation induced an alteration of the drug release profile, allowing an increase in the release of both the investigated drugs as a function of the exposure time.

In particular, for SOR-NPs at any time, the amount of sorafenib released was approximately 1.5 times higher after 5 min exposure cycles and approximately 3 times higher after 20 min exposure cycles, compared to the non-irradiated nanosystems. A different behavior was observed for LEN-NPs, as the 5 min exposure cycles do not appear to have a significant effect on the previous incubation times, but only after 8 h, in which the amount of lenvatinib released increased by about 1.5 times compared to the non-irradiated nanosystems. In contrast, after the 20 min exposure cycles at any time, the amount of lenvatinib released doubled compared to the release profile obtained without the aid of irradiation. This remote-controlled drug release would allow us to trigger and localize the release of the drug payload in a completely exclusive way at the site of action, thus minimizing the side effects.

To further study the actual thermal rise and fall during the experiment, temperatures of the external medium were recorded during and after the laser irradiation (Figure 6c). Specifically, the irradiation cycles allowed to increase the temperature as a function of the exposure time, recording a ΔT of 5 °C and 17 °C after exposures of 5 and 20 min, respectively.

Furthermore, it is interesting to note that each nanosystem kept its photothermal conversion properties unaltered after repeated laser treatments, showing the same photothermal behavior during the experiment.

### 3.5. Erythrocompatibility Studies

The evaluation of the erythrocyte compatibility of the nanosystems was performed by evaluating the hemoglobin released after their incubation with a suspension of red blood cells.

**Figure 8:**
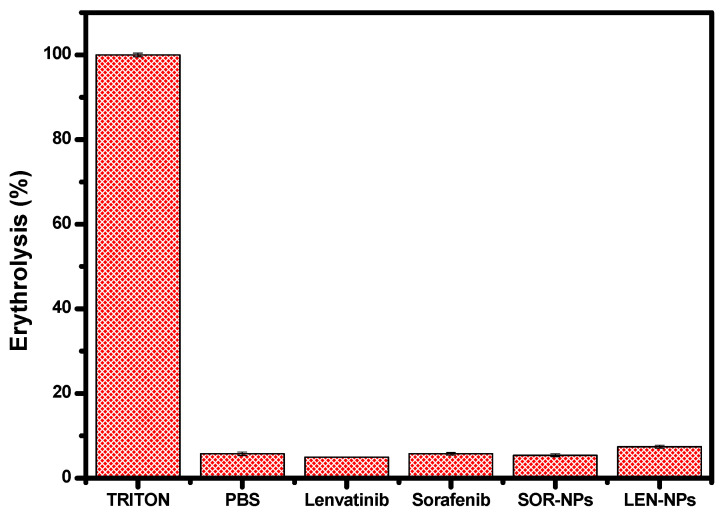
Erythrocompatibility studies. Erythrolysis percentages of SOR-NPs, LEN-NPs, equivalent amounts of free drugs, and PBS only, compared with Triton X-100 as a positive control.

As shown in Figure 8, both drug-loaded nanoparticles were highly compatible, as the released hemoglobin was less than 6% of the positive control (Triton X-100) and comparable to that released from the erythrocytes incubated with PBS only.

### 3.6. Anticancer Activity of the SOR-NPs and LEN-NPs with and without NIR Exposure

Having established that the nanoparticles obtained hold appropriate physicochemical characteristics for the drug delivery, the in vitro activity against liver cancer was evaluated. Specifically, the effect of the SOR-NPs and LEN-NPs treatment have been tested on two different hepatocarcinoma cell lines (HepG2 and HuH7) and compared to healthy cells (NHDF).

**Figure 9:**
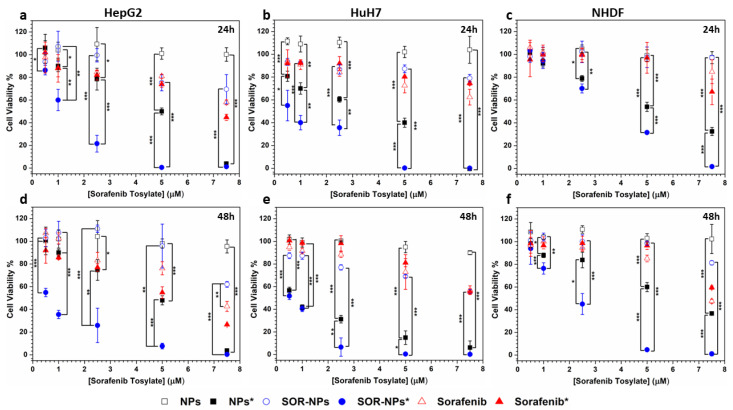
SOR-NPs dual-mode cytotoxicity studies on HepG2 (panel (**a**,**d**)), HuH7 (panel (**b**,**e**)) and NHDF (panel (**c**,**f**)) after 24 h (panel (**a**–**c**)) and 48 h (panel (**d**–**f**)) of incubation with different samples (*: NIR laser treated) (* *p* < 0.05, ** *p* < 0.01, *** *p* < 0.005).

Our results showed that the SOR-NPs on HepG2, after 24 h, have a cytotoxic effect similar to free sorafenib (Figure 9a), while a lower efficiency was observed after 48 h (Figure 9d). This effect is probably due to the slow-release profile observable without irradiation. On the other hand, after irradiation, both empty and loaded NPs at the highest concentration induced 100% of the cell death (Figure 9). Although both empty and loaded laser-irradiated NPs resulted to be effective in lowering the cell viability in a dose-dependent manner, these NPs display visible differences in their efficacy. For example, irradiated SOR-NPs corresponding to a sorafenib concentration of 2.5 μM resulted in 80% of cell death, while the equivalent amount of free sorafenib or empty NPs (laser-treated) showed a reduction in cell viability by about 20%. These data underline the synergistic effect between photothermal therapy and chemotherapy, recording for SOR-NPs an activity 4- and 3.5-fold higher after 24 h and 48 h, respectively (Figure 9a,d).

Similar data were also found for the HuH7 cell line, except for the free drug efficacy that resulted in being slightly lower for both the analyzed times (Figure 9b,e), with a 40% cell viability reduction at the higher concentration after 48h. The same result was obtained for non-irradiated SOR-NPs after 48 h. 

The data obtained on healthy cells (fibroblast cells) have shown to have advantageous features. In particular, the trend of the dose–response curves of empty and sorafenib-loaded NPs (laser-treated) showed that at lower concentrations than 2.5 μM, both the NPs were cytocompatible (around 80% cell viability) (Figure 9f), while at the same concentrations, a marked cytotoxic effect was detected on the tumor cell lines (65% and 60% for HepG2 and HuH7, respectively) (Figure 9d,e). These results, combined with the possibility of obtaining active targeting, may suggest the use of the proposed systems at low concentrations in order to obtain a selective effect against tumor cells.

**Figure 10:**
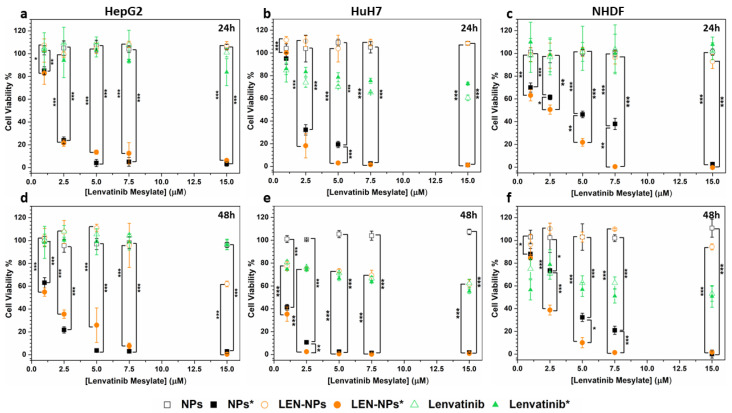
LEN-NPs dual-mode cytotoxicity studies on HepG2 (panel (**a**,**d**)), HuH7 (panel (**b**,**e**)) and NHDF (panel (**c**,**f**)) after 24 h (panel (**a**–**c**)) and 48 h (panel (**d**–**f**)) of incubation with different samples(*: NIR laser treated) (* *p* < 0.05, ** *p* < 0.01, *** *p* < 0,005).

Cytotoxicity studies involving lenvatinib mesylate, on the other hand, show a lack of efficacy of the free drug on the HepG2 cell line, registering vitality percentages always above 80% regardless of the concentration and incubation time (Figure 10a,d). Differently, lenvatinib mesylate, as well as LEN-NPs, showed a discrete cytotoxic effect on the HuH7 cell line (Figure 10b,e). Although the free drug resulted in being more active than LEN-NPs during the first 24 h of the treatment, after 48 h, their cytotoxic effects were comparable. The delayed effect of loaded NPs could be explained with the prolonged release of the drug from the nanostructure. However, on both tumor cell lines, empty NPs and LEN-NPs after a laser treatment did not show any significant cytotoxic difference, suggesting that cell death was primarily due to the hyperthermic effect. In addition, laser-treated LEN-NPs were able to induce a dose-dependent cell viability reduction in healthy cells, showing that lower concentrations affect the cell metabolism less than the tumor cell lines (Figure 10c,f). In light of the obtained results, we can state that these hybrid nanoparticles are able to induce a dose-dependent cytotoxic effect, after a laser irradiation, with or without a drug incorporation. Instead, empty non-irradiated nanosystems were cytocompatible regardless of the cell line, incubation time, and concentration used. 

### 3.7. Cell Uptake Studies

To evaluate the ability of nanoparticles to effectively enter cells, the cellular uptake was investigated by fluorescence microscopy.

To this end, empty NPs (containing AuNRs-C_12_) labelled with rhodamine B were added on both HCC and human fibroblast cell lines and analyzed after 4 and 24 h of incubation. In order to understand if the uptake was affected by the presence of the galactose target, we also performed this study pre-incubating free galactose on the cells, before adding labelled NPs, aiming the saturation of the asialoglycoprotein receptor.

**Figure 11:**
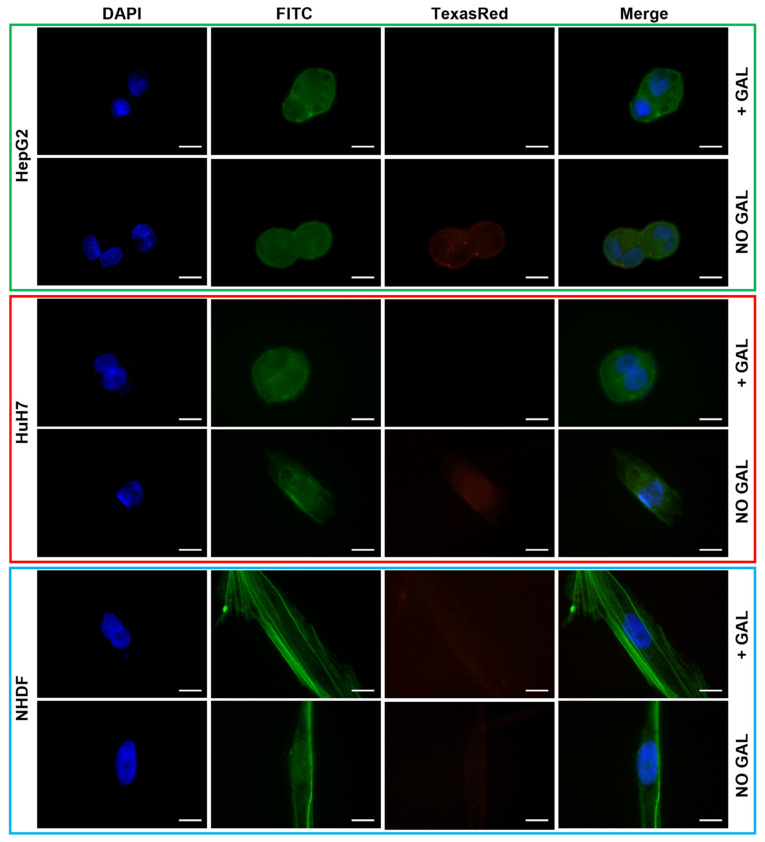
Uptake studies on hepatocarcinoma cell lines and fibroblasts after 4 h incubation with labelled drug empty NPs containing AuNRs-C_12_. Scale bar 10 μm.

**Figure 12:**
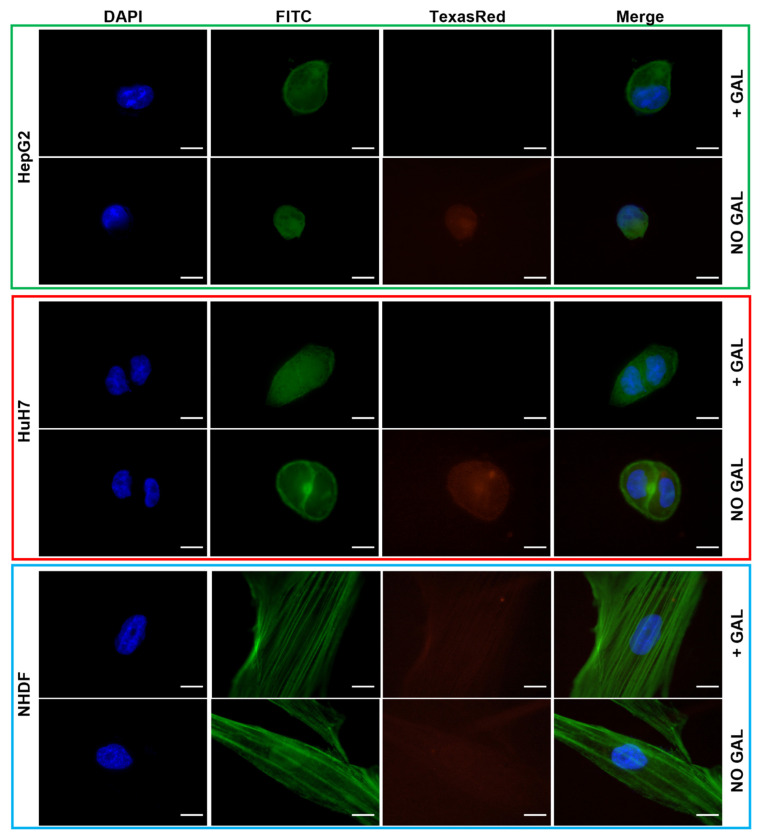
Uptake studies on hepatocarcinoma cell lines and fibroblasts after 24 h incubation with labelled drug empty NPs containing AuNRs-C_12_. Scale bar 10 μm.

The fluorescence images acquisition revealed that the nanosystems were efficiently internalized in a time-dependent fashion, with a diffuse pattern that suggests a non-specific localization throughout the cell cytosol. In particular, images acquired after 4 h of incubation (Figure 11) showed a clearly lower red fluorescence intensity compared to 24 h (Figure 12), indicating that the internalized NPs amount inside the cell increases overtime. Interestingly, the images acquired from the cells pre-incubated with free galactose showed a clear difference in the cellular uptake compared to those without a pre-incubation. The presence of free galactose appeared to drastically decrease the internalization of the NPs on both the tumor cell lines.

These findings prove that the galactose present on the NPs surfaces is crucial to improve the cellular uptake, enabling a more efficient therapy. On the contrary, the data obtained on the fibroblast cells showed a lower cellular uptake which was time-dependent, but not reliant on the presence of galactose.

Taking together these data, proposed nanoparticles can be considered virtuous candidates for an efficient and selective dual-mode hepatocarcinoma therapy.

## 4. Conclusions

In this work, we designed NIR-responsive hybrid nanocomposites, consisting of an amphiphilic polyhydroxyaspartamide-based graft copolymer (PHEA-g-BIB-pButMA)-g-PEG-GAL embedding hydrophobic gold nanorods (AuNRs-C12). This hybrid nanocomposite was designed to allow a galactose-mediated active targeting towards hepatic cells overexpressing the ASGPR and to achieve an efficient loading along with the on-demand local release of high amounts of hydrophobic anticancer drugs (sorafenib and lenvatinib) via NIR-light stimuli. The surface coating of AuNRs with 12-mercaptododecanoic acid with a coating density of about 3 chain/nm2 granted the hydrophobization of AuNRs (AuNRs-C12), conferring the dispersibility and stability in organic solvents. Then, the AuNRs-C12 obtained were incorporated inside the hydrophobic core of the nanoparticles PHEA-g-BIB-pButMA-g-PEG-GAL, giving rise to the hybrid nanosystems SOR-NPs and LEN-NPs, with a diameter of about 214 nm and 148 nm, respectively, endowed with a top-notch NIR photothermal conversion, high drug loading, and NIR-driven enhancement of the drug release. The high biocompatibility of these new carriers, as well as the anticancer effect of drug-loaded nanosystems with and without an NIR exposure, were assessed by an in vitro MTS assay on both HCC and healthy human cell lines. In addition, the fluorescence microscopy analyses demonstrated that these new hybrid nanocomposites were able to enter into the hepatic cancer cells overexpressing the ASGPR, where they can potentially release heat and drugs. These results indicate that the proposed hybrid nanocomposites can be considered to be promising candidates for the chemo-photothermal targeted therapy of solid tumors. This innovative approach has the advantage to potentially overcome multi-drug resistance in cancer, developing a multimodal tool able to selectively recognize and kill cancer cells by dual-mode therapy.

## Figures and Tables

**Table 1:** Values of weight-average molar weight (Mw) and polydispersity (Mw/Mn) determined through SEC analysis and molar derivatization degrees (DD %) calculated by ^1^H-NMR analysis.

COPOLYMERS	${\bar{M}}_{n}$	${\bar{M}}_{W}$	${\bar{M}}_{W}/{\bar{M}}_{n}$	DD_BIB_%	DDB_utMA_%	DD_PEG_%
PHEA	32,461	45,446	1.4	-	-	-
PHEA-g-BIB	39,524	67,192	1.7	35	-	-
PHEA-g-BIB-PButMA	210,051	399,098	1.9	35	385	-
PHEA-g-BIB-PButMA-g-PEG-GAL	270,299	540,599	2.0	35	385	7

**Table 2:** Characterization of SOR-NPs and LEN-NPs: dynamic light scattering data, surface charge, drug, and gold content.

	Z-Average (nm)	PDI	Z-Potenzial (mV)	Drug Loading (% *w*/*w*)	Entrapment Efficiency (% *w*/*w*)	Au Loading (% *w*/*w*)
SOR-NPs	214.3 ± 3.4	0.28 ± 0.1	−10.8 ± 7.9	9.0 ± 0.2	96.6 ± 2.1	7.6 ± 0.3
LEN-NPs	148.1 ± 11.1	0.34 ± 0.1	−15.2 ± 7.2	5.4 ± 0.2	58.4 ± 2.9	8.0 ± 0.1

## Data Availability

Not applicable.

## Supplementary Materials

The following supporting information can be downloaded at: https://www.mdpi.com/article/10.3390/pharmaceutics14112503/s1, Figure S1: ^1^H-NMR spectrum of PHEA-g-BIB graft copolymer (DMF-d_7_); Figure S2: ^1^H-NMR spectrum of PHEA-g-BIB-pButMA graft copolymer (DMF-d_7_); Figure S3: ^1^H-NMR spectrum of PHEA-g-BIB-pButMA-g-PEG-GAL graft copolymer (DMF-d_7_); Figure S4: Differential Scanning Calorimetry of sorafenib tosylate (black), PHEA-g-BIB-pButMA-g-PEG-GAL (red), and SOR-NPs (blue); Figure S5: Differential Scanning Calorimetry of lenvatinib mesylate (black), PHEA-g-BIB-pButMA-g-PEG-GAL (red), and LEN-NPs (blue).
