# Supplementary material for: Galactosylated Polymer/Gold Nanorods Nanocomposites for Sustained and Pulsed Chemo-Photothermal Treatments of Hepatocarcinoma
Source: Pharmaceutics. 2022 Nov 18;14(11):2503. doi: 10.3390/pharmaceutics14112503 (PMC9696514; doi:10.3390/pharmaceutics14112503)
Supplement: Supplementary file 1 [file pharmaceutics-14-02503-s001.zip › pharmaceutics-2012570-supplementary.pdf]

Supporting information

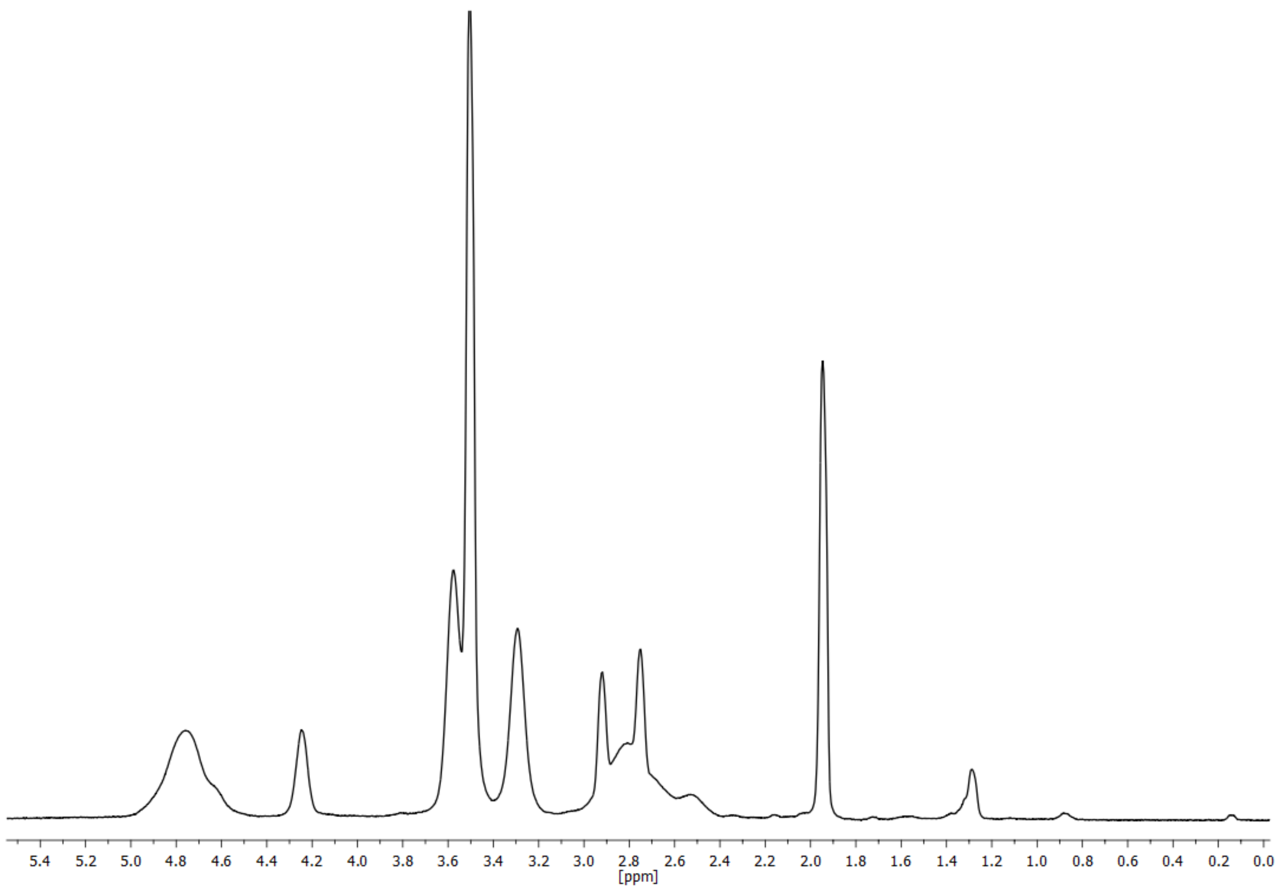

Figure S1. ^{1}H-NMR spectrum of PHEA-g-BIB graft copolymer (DMF-d_7)

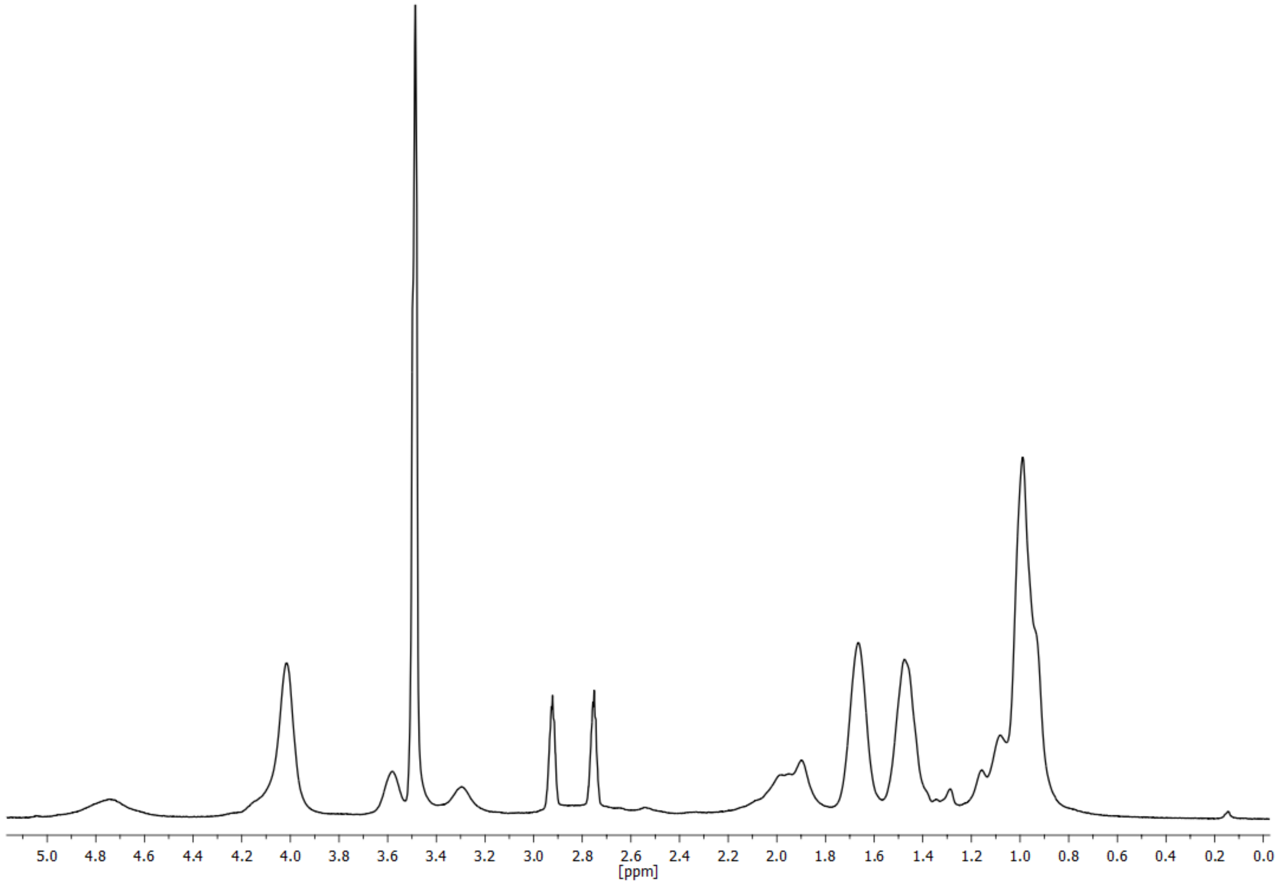

Figure S2. ^{1}H-NMR spectrum of PHEA-g-BIB-pButMA graft copolymer (DMF-d_7)

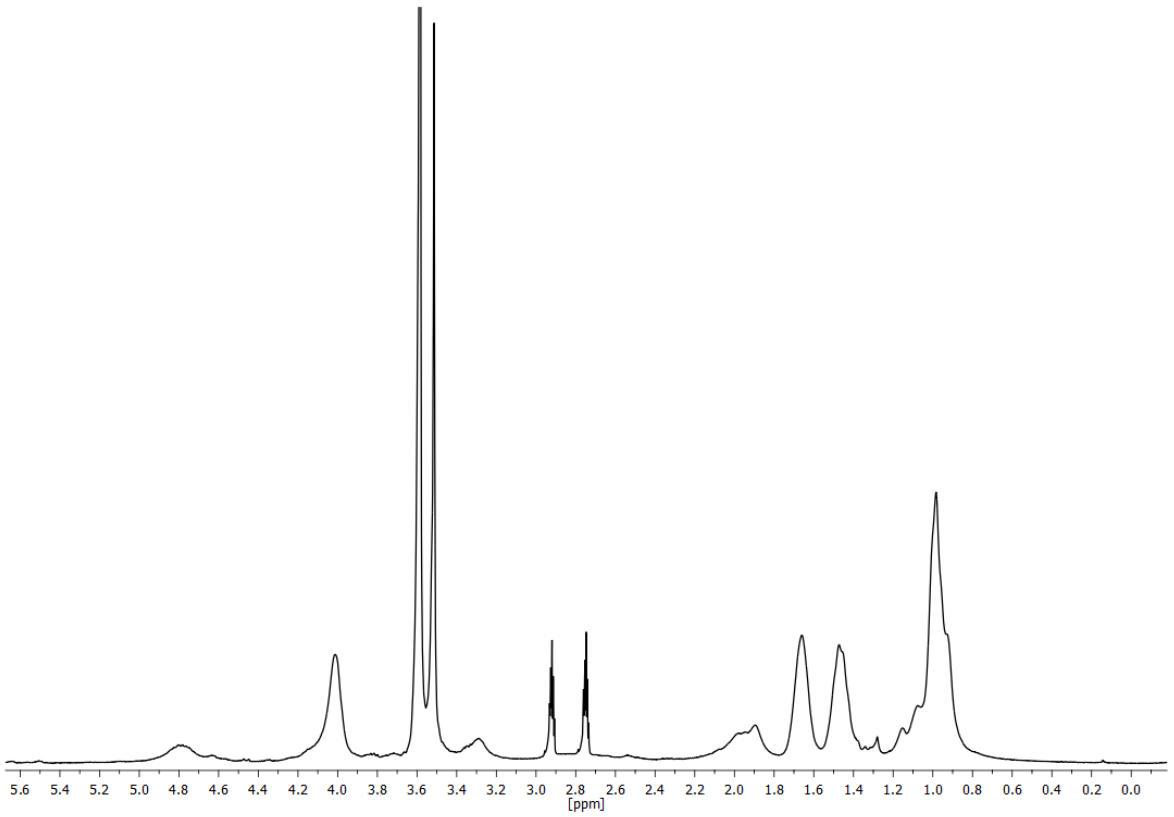

Figure S3. ^{1}H-NMR spectrum of PHEA-g-BIB-pButMA-g-PEG-GAL graft copolymer (DMF-d_7)

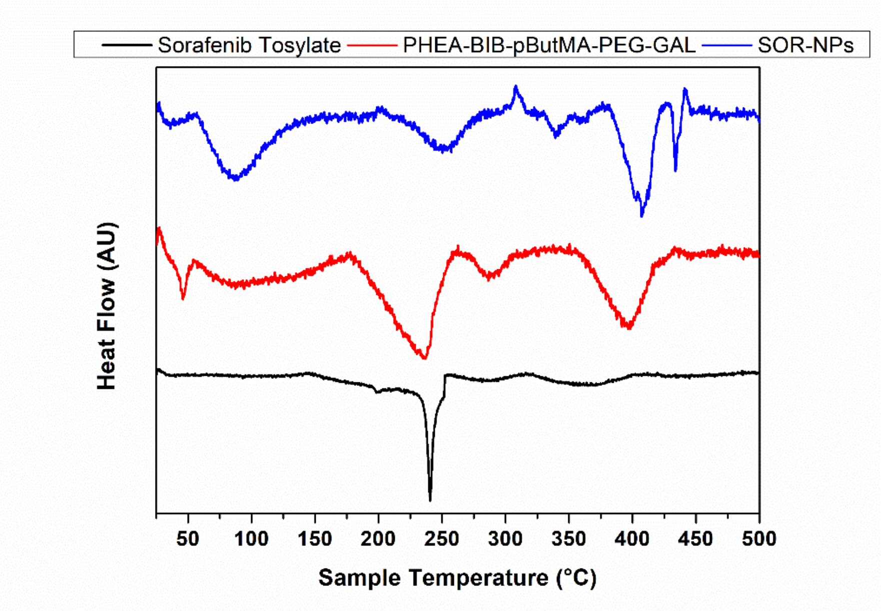

Figure S4. Differential Scanning Calorimetry of sorafenib tosylate (black), PHEA-g-BIB-pButMA-g-PEG-GAL (red), and SOR-NPs (blue)

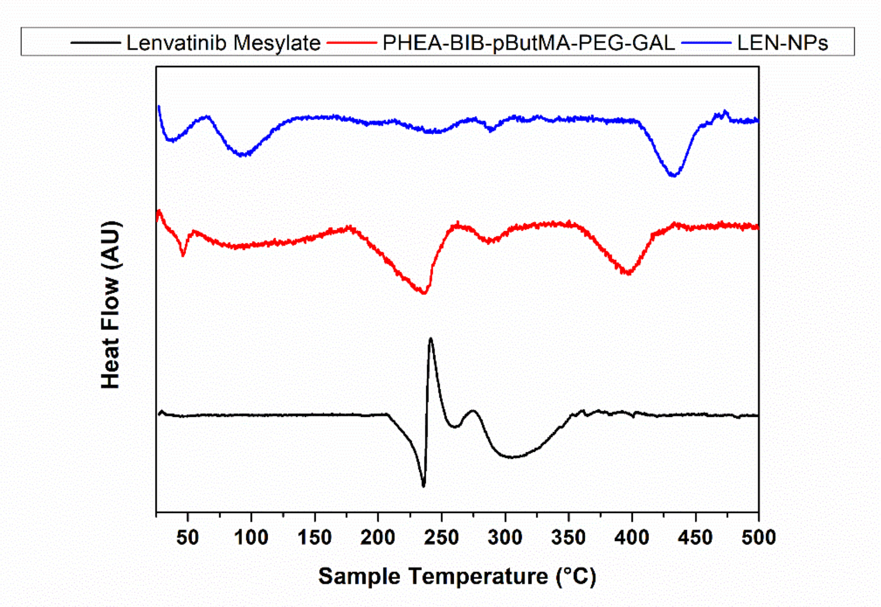

Figure S5. Differential Scanning Calorimetry of lenvatinib mesylate (black), PHEA-g-BIB-pButMA-g-PEG-GAL (red), and LEN-NPs (blue)
